# Pigmented Purpuric Dermatosis in a Kickboxer: The Role of Repetitive Microtrauma

**DOI:** 10.7759/cureus.79196

**Published:** 2025-02-17

**Authors:** Riley Shin, Sanjana Likki, Troy A Black, Rashid M Rashid

**Affiliations:** 1 School of Medicine, Texas Tech University Health Sciences Center, Lubbock, USA; 2 School of Medicine, University of Texas Health Science Center at Houston McGovern Medical School, Houston, USA; 3 Dermatology, Mosaic Dermatology, Houston, USA

**Keywords:** capillaritis, capillary fragility, hemosiderin, pigmentation disorders, pigmented purpuric dermatosis, vascular diseases

## Abstract

A 41-year-old male kickboxer presented with bilateral, macular, red skin discoloration around the ankles, fading proximally and exhibiting a sharp linear demarcation above the lateral malleoli. The discoloration was more pronounced medially and on the right side. Histology revealed spongiosis, intraepidermal and papillary dermal erythrocytes, and perivascular lymphocytic inflammation, confirming the diagnosis of pigmented purpuric dermatosis (PPD). Repetitive microtrauma from kickboxing likely contributed to capillary fragility, compounded by exercise-induced inflammation and increased venous pressure, which may have promoted endothelial dysfunction and capillary rupture. PPD, or capillaritis, refers to a group of benign, chronic dermatologic conditions characterized by purpuric eruptions such as macules, patches, and petechiae, often idiopathic in nature. This case highlights the potential role of physical activity in triggering PPD. It emphasizes the importance of recognizing exercise-related patterns to refine diagnosis and management strategies for patients involved in high-impact sports.

## Introduction

Pigmented purpuric dermatosis (PPD), or capillaritis, encompasses a group of benign, chronic, and generally asymptomatic dermatologic conditions characterized by purpuric eruptions, including red to purple macules, patches, and petechiae. These lesions result from red blood cell leakage (erythrocyte extravasation) and the subsequent deposition of hemosiderin, leading to progressive color changes that often appear as red-brown or golden-brown discolorations [1]. While the exact etiology of PPD remains unclear, contributing factors may include poor blood circulation (venous stasis), exercise, capillary fragility, immune-related inflammation, and certain medications. Although often idiopathic, PPD can present diagnostic challenges due to its varied manifestations and potential associations with systemic or localized triggers [2]. A notable yet underrecognized trigger is mechanical stress, particularly repetitive trauma from physical activity. We report a case of PPD in a kickboxer, highlighting a distinct clinical pattern associated with high-impact, exercise-induced microtrauma, which broadens our understanding of how physical activity may contribute to its pathogenesis.

## Case presentation

**Figure 1:**
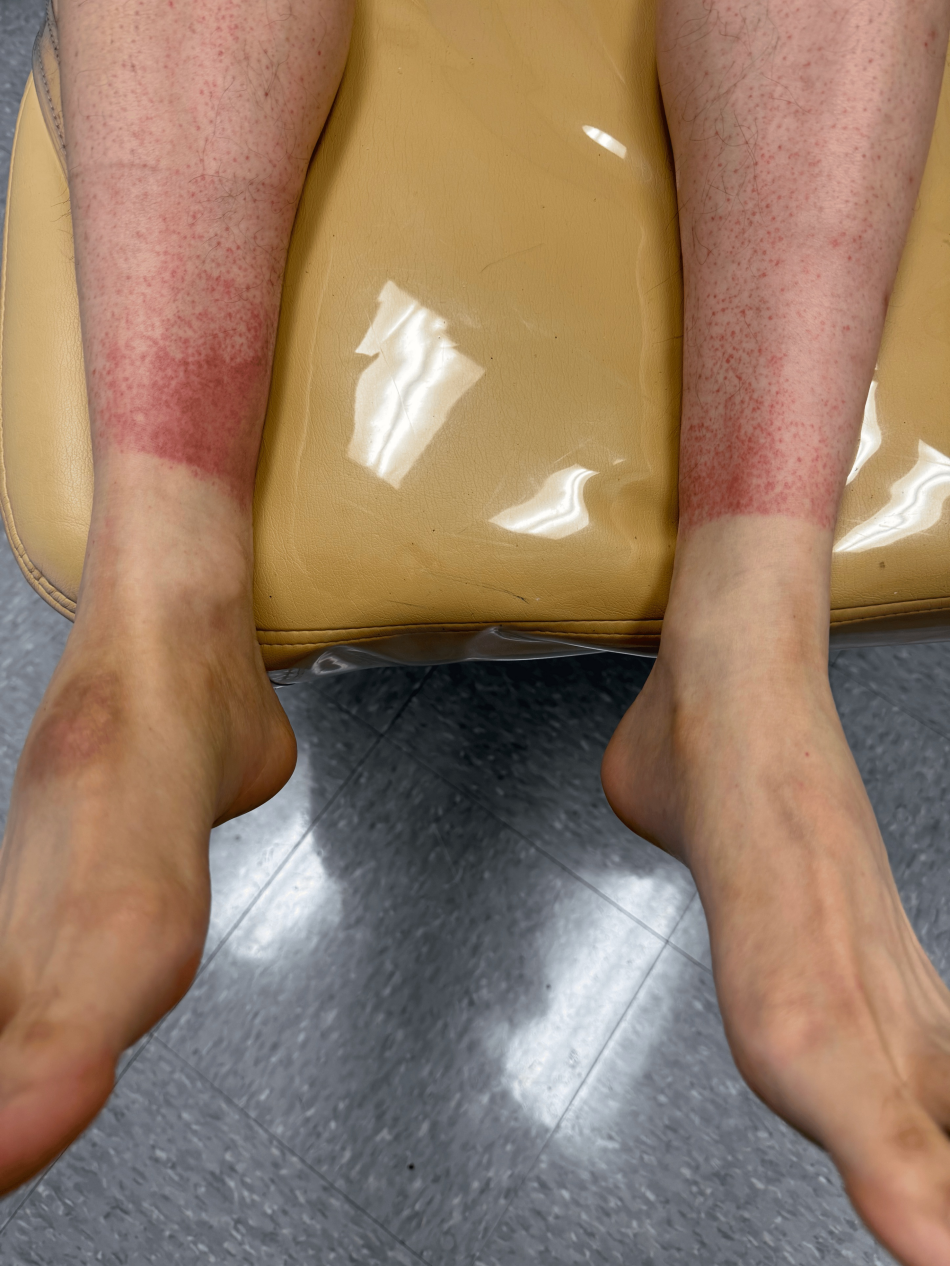
Pigmented purpuric dermatosis was pronounced predominantly on the medial aspect of both ankles, with greater severity on the patient’s right side

In July 2024, a 41-year-old male kickboxer with a 10-year history in the sport presented with bilateral, macular, red discoloration around the ankles. The patient reported that the discoloration developed gradually over 3 to 4 days following an intensive training session and was more pronounced on the medial aspects of both ankles. The lesions exhibited a sharp, circumferential linear demarcation above the malleolus (Figure 1), fading as they ascended the legs. He denied associated symptoms such as pain, itching, or swelling. There were no systemic complaints, including fever, weight loss, or fatigue.

The patient’s medical history was unremarkable, with no history of coagulopathy, venous insufficiency, or other dermatologic conditions. He was not taking any medications or supplements and had no known allergies. He denied any history of recent travel, infections, or exposure to new topical products. A thorough review of systems and family history revealed no significant findings.

On physical examination, the macular erythematous lesions were non-blanching and exhibited a uniform red to reddish-brown appearance. They showed medial prominence with more pronounced involvement on the right ankle, consistent with the patient’s dominant side. The remainder of the physical examination, including other skin surfaces, was normal. Dermoscopy revealed punctate reddish areas without evidence of vascular anomalies, erosions, or ulceration.

**Figure 2:**
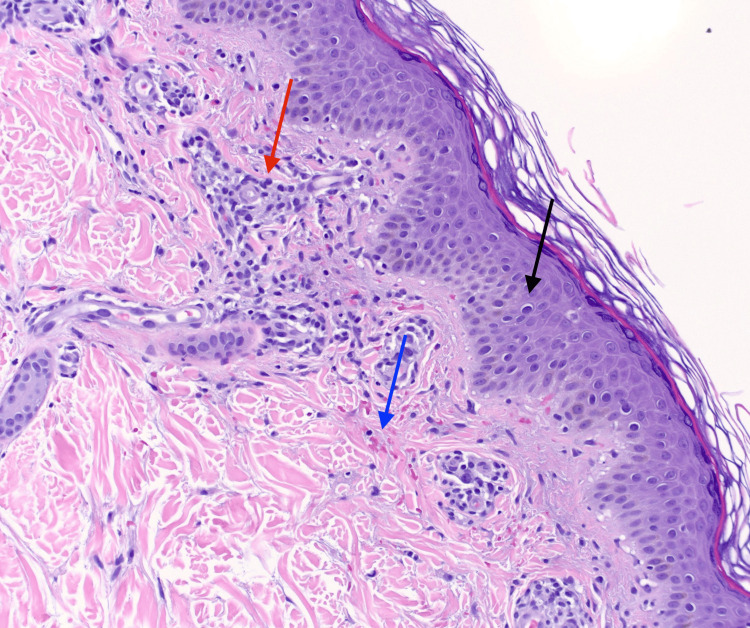
Hematoxylin and eosin histologic preparation shown at 20x magnification displaying spongiosis (black arrow), intraepidermal and papillary dermal erythrocytes (blue arrow), and perivascular lymphocytic inflammation (red arrow)

Histologic examination of a lesional biopsy demonstrated spongiosis, intraepidermal and papillary dermal extravasated erythrocytes, perivascular lymphocytic inflammation, and hemosiderin deposition in the papillary dermis (Figure 2). These findings confirmed the diagnosis of PPD.

Further laboratory workup, including complete blood count, coagulation panel, and inflammatory markers, showed no abnormalities. Based on the patient’s history, clinical examination, and histopathologic findings, PPD was attributed to repetitive microtrauma from high-impact exercise during kickboxing. Given the absence of symptoms and the self-limiting nature of the condition, conservative management with reassurance and observation was recommended.

## Discussion

Histologically, PPD is characterized by perivascular lymphocytic infiltration, extravasated erythrocytes, hemosiderin deposition, and dilated capillaries with endothelial swelling. While the various subtypes of PPD share this underlying pathologic mechanism, they are further classified based on differences in lesion shape, arrangement, associated symptoms, and additional features like lichenoid changes or telangiectasia. This case most closely resembles Schamberg’s disease, the most common subtype of PPD, recognized by its characteristic “cayenne pepper-like” appearance, consisting of chronic, progressive red-brown patches that coalesce with generally no other associated symptoms [2]. However, the sharp linear boundary above the malleolus and medial prominence observed are notably distinct. These findings suggest a localized pattern likely influenced by repetitive microtrauma, setting this presentation apart from the typical features of Schamberg’s disease.

Exercise and mechanical trauma have been underrecognized contributors to the development and localization of PPD. Repetitive microtrauma from high-impact activities can increase capillary fragility, leading to capillary leakage. Exercise-induced inflammation and increased venous pressure may further exacerbate capillary rupture. A U-shaped relationship exists between exercise intensity and vascular health, where both insufficient and excessive levels of exercise can negatively impact vascular function. High-intensity exercise may also impair endothelial function by increasing levels of reactive oxygen species and decreasing levels of circulating antioxidants, further exacerbating vascular fragility [3]. Recent evidence suggests that prolonged exercise induces mitochondrial reactive oxygen species (ROS) production in human skeletal muscle, driven in part by elevated free fatty acid levels during endurance exercise. While ROS plays an adaptive role in exercise, promoting mitochondrial biogenesis and glucose uptake, excessive levels can compromise endothelial function and contribute to microvascular fragility, potentially contributing to the development of PPD in physically active individuals [4].

The medial prominence and sharp linear demarcation above the malleolus observed likely reflect localized trauma from repetitive kicking motions, which predominantly impact the medial regions of the lower extremity. Although the patient denied wearing specialized kickboxing equipment, such as sparring boots, protective gearlike ankle wraps, or socks, it may have further shaped this pattern by shielding certain regions. The predominance of lesions on the right side aligns with the patient’s right-side dominance, where stronger or more frequent kicks likely exacerbated trauma in this location.

Although microtrauma-induced PPD has not been extensively documented, existing literature supports the notion that localized trauma may lead to distinctive patterns of cutaneous lesions. For example, Grabell et al. found that among a cohort of 329 patients, 16% (53 patients) developed morphea lesions in areas of prior skin trauma [5]. Similarly, Golfer’s Vasculitis, also known as exercise-induced vasculitis, is a condition that predominantly affects healthy individuals following prolonged physical activity, such as walking or golfing. The etiology is multifactorial, involving heat-induced muscular hyperthermia, immune alterations, venous stasis, and changes in skin blood flow. Clinically, it presents with a range of skin manifestations, including erythematous rash, purpura, pseudo-urticaria, and lower extremity edema, with histological findings showing urticarial or leukocytoclastic vasculitis. While self-limiting, relapses are common, and preventive measures such as compression stockings and avoiding prolonged exercise in hot weather have been suggested [6]. Supporting this venous stasis mechanism, Laborde described a case of exercise-induced vasculitis in a hiker who developed lower leg purpura after long-distance walks with a backpack, with further rash episodes prevented by compression stockings [7]. Additionally, a case-control study by Quéneau et al. examined exercise-induced vasculitis in hikers, emphasizing the role of prolonged exercise, heat exposure, and venous stasis as contributing factors [8]. These reports further illustrate the role of mechanical stress in provoking cutaneous vascular pathology, paralleling the kickboxing-induced presentation of PPD in our patient.

Treatment guidelines for PPD are not well-defined, and the condition is often persistent and refractory. Conservative or non-pharmacologic approaches are typically appropriate for asymptomatic patients, as in this case. For symptomatic or cosmetically concerned patients, treatments such as bioflavonoids, vitamin C, topical corticosteroids, topical calcineurin inhibitors, phototherapy, and topical photodynamic therapy have been explored [9]. For example, a small pilot study demonstrated complete clearance of lesions in patients treated with oral bioflavonoids (rutoside 50 mg twice daily) and ascorbic acid (500 mg twice daily) over four weeks, with sustained improvement at three months [10]. Additionally, phototherapy, particularly narrowband UVB (NB-UVB), has shown successful treatment outcomes in six patients with PPD following 24-28 sessions of NB-UVB therapy, with additional maintenance treatments given in some cases as needed [11]. These interventions highlight the variety of approaches explored, though individualized treatment plans remain essential given the diversity of causes and treatment responses in PPD.

## Conclusions

This case highlights a unique clinical presentation of PPD, likely secondary to repetitive microtrauma. The patient’s cutaneous presentation and physical activity align with the hypothesis that high-impact exercises, like kickboxing, may exacerbate capillary fragility or increase the risk of capillary rupture, contributing to the development of PPD. Additionally, the sharp linear demarcation above the malleolus suggests a potential role of protective wear, such as socks or tight banding, in shaping this pattern. While PPD is often idiopathic, this case provides valuable insight into how physical activity can influence its presentation and severity. Conservative management remains effective in asymptomatic cases, but awareness of potential contributing factors can guide preventive measures and inform future investigations into the pathophysiology of PPD.
